# Bioinformatics-based identification of key genes and pathways associated with colorectal cancer diagnosis, treatment, and prognosis

**DOI:** 10.1097/MD.0000000000030619

**Published:** 2022-09-16

**Authors:** Chaochao Wang, Li Zhang

**Affiliations:** a Department of Emergency Medicine, The Affiliated Hospital of Southwest Medical University, Luzhou, Sichuan, 646000, China; b Health Management Center, The Affiliated Hospital of Southwest Medical University, Luzhou, Sichuan, 646000, China.

**Keywords:** bioinformatics, colorectal cancer, key genes, signaling pathways

## Abstract

Colorectal cancer (CRC) is known to display a high risk of metastasis and recurrence. The main objective of our investigation was to shed more light on CRC pathogenesis by screening CRC datasets for the identification of key genes and signaling pathways, possibly leading to new approaches for the diagnosis and treatment of CRC. We downloaded the colorectal cancer datasets from the Gene Expression Omnibus (GEO) database site. We used GEO2R to screen for differentially expressed genes (DEGs) of which those with a fold change >1 were considered as up-regulated and those with a fold change <-1 were considered as down-regulated on the basis of a *P* < .05. “Gene ontology (GO)” and “Kyoto Encyclopedia of Genes and Genomes (KEGG)” data were analyzed by the “DAVID” software. The online search tool “STRING” was used to search for interacting genes or proteins and we used Cytoscape (v3.8.0) to generate a PPI network map and to identify key genes. Finally, survival analysis and stage mapping of key genes were performed using “GEPIA” with the aim of elucidating their potential impact on CRC. Our study revealed 120 intersecting genes of which 55 were up- and 65 were downregulated, respectively. GO analysis revealed that these genes were involved in cell proliferation, exosome secretion, G2/M transition, cytosol, protein binding, and protein kinase activity. KEGG pathway analysis showed that these genes were involved in cell cycle and mineral absorption. The Cytoscape PPI map showed 17 nodes and 262 edges, and 10 hub genes were identified by top 10 degrees. Survival analysis demonstrated that the AURKA, CCNB1, and CCNA2 genes were strongly associated with the survival rate of CRC patients. In addition, CCNB1, CCNA2, CDK1, CKS2, MAD2L1, and DLGAP5 could be correlated to pathological CRC staging. In this research, we identified key genes that may explain the molecular mechanism of occurrence and progression of CRC but may also contribute to an improvement in the clinical staging and prognosis of CRC patients.

## 1. Introduction

Colorectal cancer is a very common cancer worldwide. Its mortality rate is the second highest of all cancers and is usually associated with a poor prognosis, especially in advanced cancer stages. According to World Health Organization statistics, almost 1.4 million new cases and 700,000 deaths related to CRC are reported worldwide every year.^[1,2]^ In 2015, China reported about 376,000 new cases and 191,000 deaths due to CRC.^[3]^ CRC is a multifactorial gastrointestinal disease and a huge problem faced by doctors and researchers. Relevant clinical studies have shown that the current 5-year survival rate is maintained at only around 60%^[4]^ and that this is closely related to the time of diagnosis. Therefore, early diagnosis and therapeutic intervention, as well as elucidating the underlying molecular mechanisms and the improvement of the survival rate of CRC patients remain a challenge.

A large number of high-throughput gene chip experiments have been conducted to screen for differentially expressed genes in human tumor and normal tissues which has led to a new direction of exploring molecular mechanisms of tumor development.^[5]^ With the help of bioinformatics, the huge amount of data generated from these analyses, can now comprehensively analyze key genes of disease diagnosis, treatment and prognosis. The identified key genes can then be verified by further experiments/studies. The analysis of different clinical datasets may reveal some unprecedented clues for the treatment and prognosis of tumors in general. In recent years, researchers have analyzed a series of CRC microarray datasets and discovered a large number of DEGs that are involved in e.g. cellular components or molecular functions.

This paper aims at providing a comprehensive analysis of colon cancer incidence based on genetic data of GEO, to establish a genetic network leading to a new understanding of how to recognize colon cancer. It also puts forward new strategies for the diagnosis and clinical treatment of colon cancer and therefore, the improvement of the survival rate.

## 2. Materials and methods

### 2.1. DEG filtering

The gene expression profiles GSE77953, GSE87211, GSE110223, and GSE113513 were downloaded from the GEO database. We used GEO 2R to identify DEGs within these sample groups. Data with a *P* < .05 were considered as statistically significantly different and those genes showing a log2FC > 1 were considered as upregulated, whereas those genes showing a log2FC < −1 were considered as downregulated.

### 2.2. GO and KEGG pathway analysis

Both, GO and KEGG analysis help to better understand the mechanisms associated with disease progression. The Database for Annotation, Visualization and Integrated Discovery (DAVID) is an online bioinformatics-based network that is routinely applied for the annotation of gene and protein functions.^[6]^ The screening results, comprising the common genes, were fed into DAVID for GO and KEGG analysis with *P* < .05 as the selection criterion. The top ten BP, MF, CC, and KEGG results were visualized by creating bubble plots.

### 2.3. Screening of key genes

The STRING website aims at integrating all known and predicted associations between proteins, like physical interactions and functional associations.^[7]^ DEGs were mapped by STRING to assess an interlinkage between the genes and defined a confidence score of > 0.4 as significant. Cytoscape is a network biology analysis and visualization software^[8]^ that was used to generate PPI networks. The Cytoscape plugin Molecular Complex Detection (MCODE) was used to detect deeper connected genes within the PPI network. Finally, according to the degree levels determined by CytoHubba, the top 10 ranked genes were considered as key genes.

### 2.4. Validating key genes

The Gene Expression Profiling Interactive Analysis (GEPIA) website is a non-commercial platform providing access to a gene interaction analysis tool that can be customized with desired features including differential expression analysis, patient survival analysis, and similar genetic assays.^[9]^ We employed the box plot to visualize mRNA expression and the violin chart to display the expression of key genes at different stages of CRC.

### 2.5. Survival analysis

Similarly, to validate the role of key genes in patient prognosis, we applied the GEPIA database to obtain patient overall survival data. Log P-values and 95% confidence intervals are shown in the figure.

### 2.6. Ethics statement

Our data are all from public databases for biological analysis, there’s no direct contact with patients and animals, thus there’s no need for ethical review.

## 3. Results

### 3.1. DEG identification

**Figure 1.**
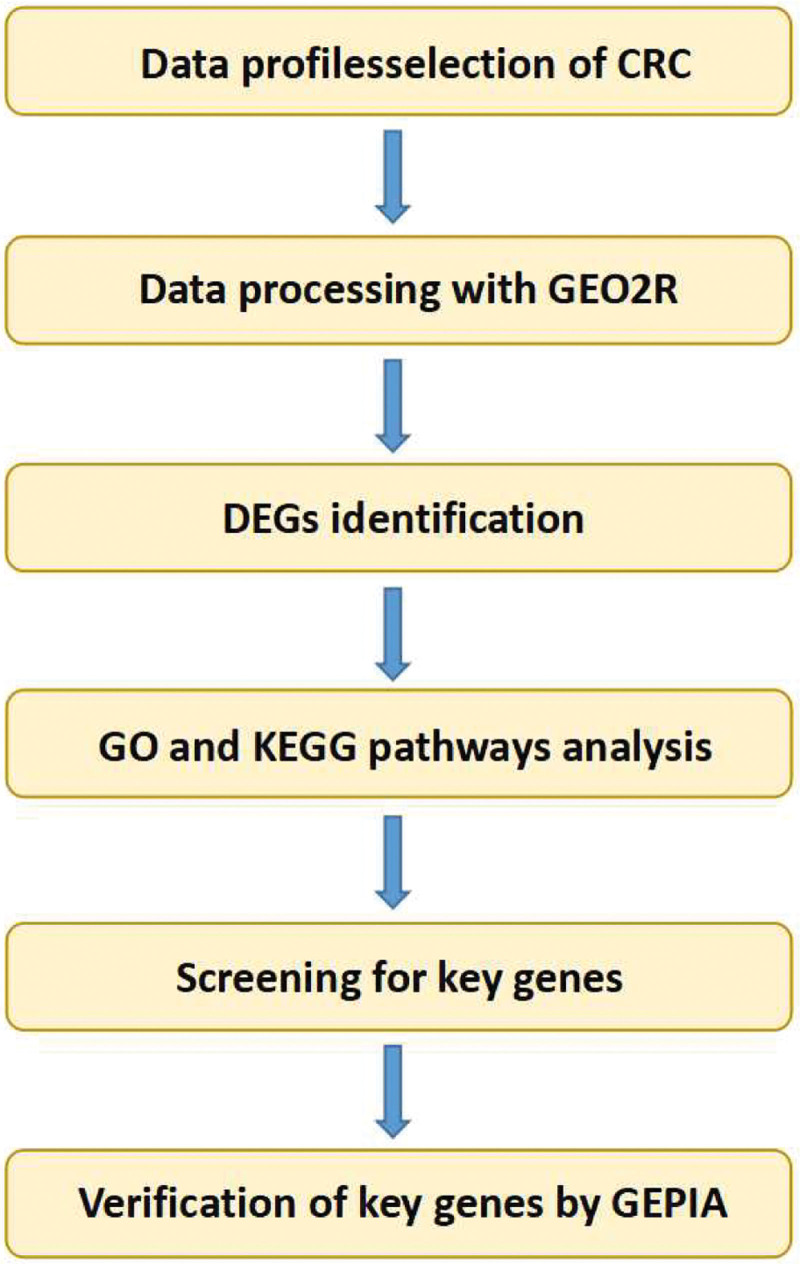
The flowchart of the data analysis.

**Figure 2.**
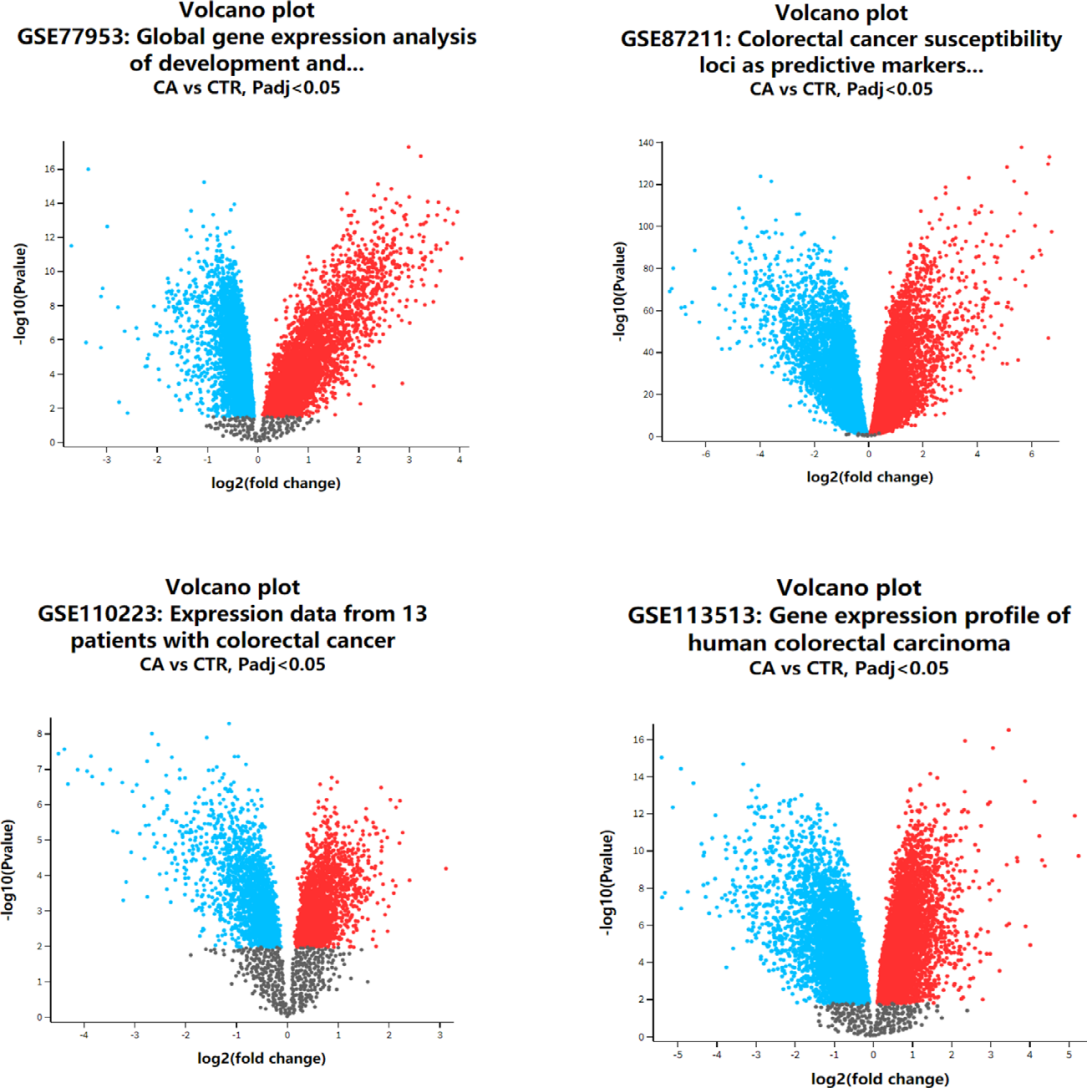
Volcano plot of detected genes. Blue dots represent down-regulated genes; Red dots represent up-regulated genes; Black dots represent genes with no difference in expression.

**Figure 3.**
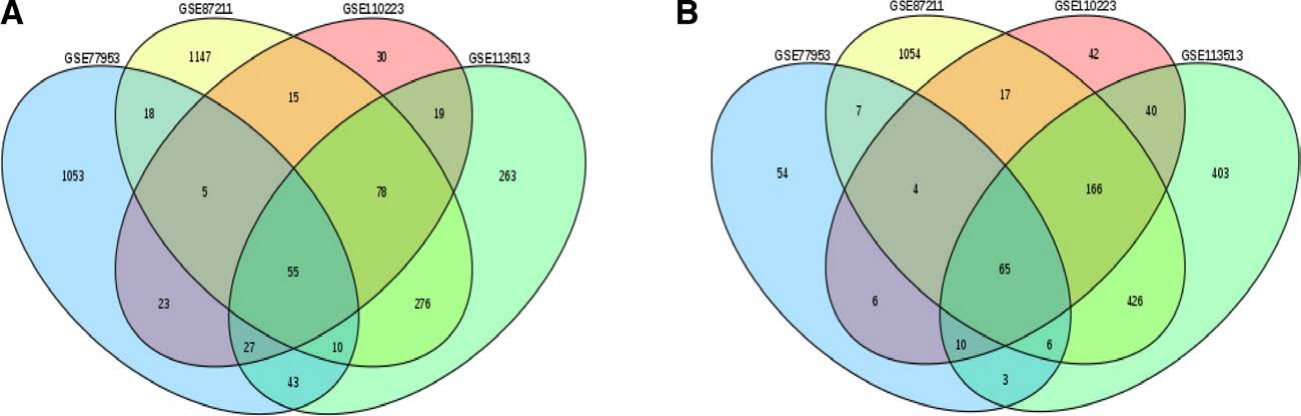
Venn diagrams of all DEGs including 55 upregulated (A) and 65 downregulated (B) genes that overlap within the 4 datasets.

In this study, key genes and their biological function were identified based on integrated bioinformatics analysis (Fig. 1). Genetic expression profiles GSE7953, GSE87211, GSE10223, and GSE13513 were selected as the database. A total of 200 normal samples and 258 cancer patient samples contained in 4 datasets were analyzed by GEO 2R (Fig. 2). There were 120 common DEGs, including 55 upregulated and 65 downregulated genes contained in the 4 datasets (Fig. 3)

### 3.2. GO and KEGG pathway analysis

**Figure 4.**
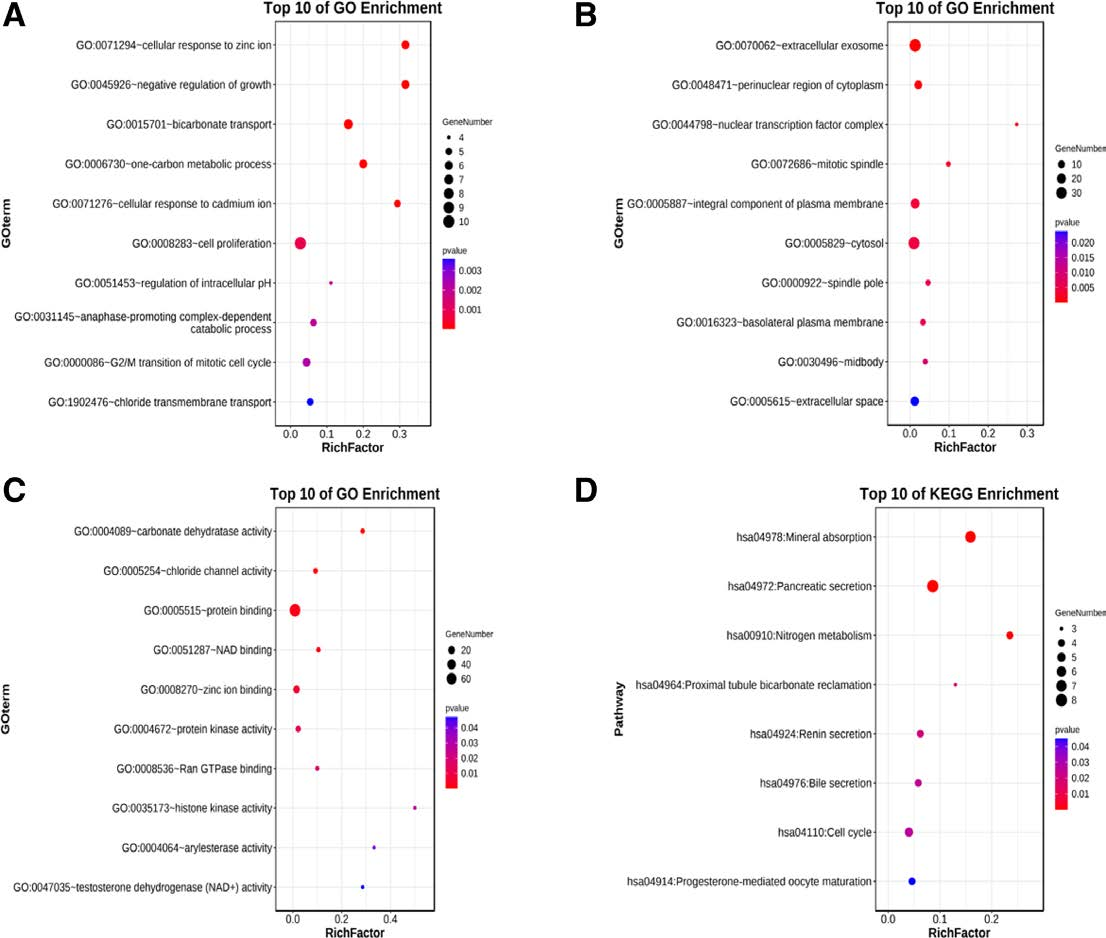
GO and KEGG pathway analysis of CRC-related genes. The enriched GO terms in the biological process (BP) group; (B) the cellular component (CC) group; (C) the molecular function (MF) group; (D) the KEGG signaling pathway.

To explore the possibility of improving and reducing the level of general DEG biological function, we used the DAVID website for enrichment analysis through GO and KEGG. The identified genes could be stratified into different groups belonging to biological processes (BP), including cell proliferation and G2/M transition of the mitotic cell cycle. The cellular component (CC) group comprising among others, extracellular exosome and cytosol. The binding and kinase activity of proteins belong to the molecular function (MF) group. KEGG pathway analysis showed that DEGs mainly occurred in the cell cycle and mineral absorption group (Fig. 4).

### 3.3. PPI network and key gene screening

**Figure 5.**
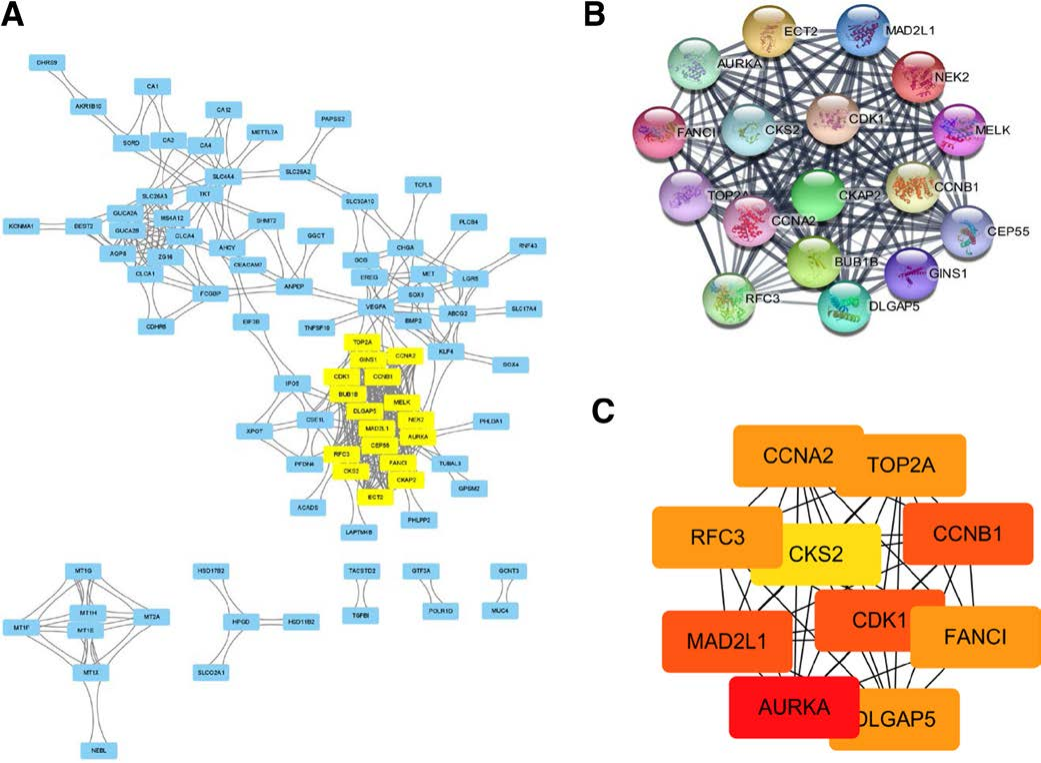
Screening results of the top 10 identified key genes. (A) PPI network consisted of 17 nodes and 262 edges of overlapping DEGs. (B) The interaction network of the 17 key genes. (C) Top 10 key genes identified by CytoHubba of Degree plug-in.

The String website was used to create PPI connections to evaluate the interaction between common DEGs. By using the MCODE plug-in of Cytoscape, a PPI network comprising 17 nodes and 262 edges was generated (Fig. 5A–B). Cytohubba identified the top 10 Degree levels of genes in the network (Table 1, Fig. 5C). AURKA: aurora kinase A, CDK1: cyclin-dependent kinase 1, FANCI: FA complementation group I, MAD2L1: mitotic arrest deficient 2 like1, DLGAP5: DLG associated protein 5, CCNB1: cyclin B1, TOP2A: DNA topoisomerase II alpha, RFC3: replication factor C subunit 3, CCNA2: cyclin A2, and CKS2: cyclin-dependent kinases regulatory subunit 2. We achieved GO and KEGG enrichment analysis of 10 key genes by DAVID which revealed they were mainly taking part in “cell division”, “cell proliferation”, “protein binding”, “nucleus” and “Cell cycle”(Table 2).

**Table 1:** The top 10 degrees of key genes.

Gene name	Degree score	Expression
AURKA	40	UP
CDK1	36	UP
MAD2L1	36	UP
CCNB1	36	UP
FANCI	34	UP
TOP2A	34	UP
CCNA2	34	UP
DLGAP5	34	UP
RFC3	34	UP
CKS2	32	UP

**Table 2:** GO and KEGG pathway enrichment analysis of 10 key genes.

	**Term**	***P* value**	**Genes**
	GO:0051301~cell division	4.50E-07	CCNA2, CCNB1, CDK1, CKS2, MAD2L1, AURKA
	GO:0007095~mitotic G2 DNA damage checkpoint	2.67E-05	CCNA2, FANCI, CDK1
	GO:0042787~protein ubiquitination involved in ubiquitin-dependent protein catabolic process	5.98E-05	CCNB1, CDK1, MAD2L1, AURKA
**BP**	GO:0006977~DNA damage response, signal transduction by p53 class mediator resulting in cell cycle arrest	4.75E-04	CCNB1, CDK1, AURKA
	GO:0051437~positive regulation of ubiquitin-protein ligase activity involved in regulation of mitotic cell cycle transition	7.13E-04	CCNB1, CDK1, MAD2L1
	GO:0000086~G2/M transition of mitotic cell cycle	.00229126	CCNB1, CDK1, AURKA
	GO:0007067~mitotic nuclear division	.007303634	CCNA2, CDK1, AURKA
	GO:0008283~cell proliferation	.015412716	CDK1, CKS2, DLGAP5

	**Term**	***P* value**	**Genes**
	GO:0019901~protein kinase binding	8.33E-04	CCNA2, CCNB1, CKS2, AURKA
	GO:0035173~histone kinase activity	.002131059	CCNB1, CDK1
**MF**	GO:0005515~protein binding	.002794077	TOP2A, CCNA2, FANCI, CCNB1, RFC3, CDK1, CKS2, DLGAP5, MAD2L1, AURKA
	GO:0003682~chromatin binding	.017297176	TOP2A, CDK1, CKS2
	GO:0004693~cyclin-dependent protein serine/threonine kinase activity	.017985763	CCNB1, CDK1
	GO:0043130~ubiquitin binding	.040834351	TOP2A, CKS2

	**Term**	***P* value**	**Genes**
	GO:0072686~mitotic spindle	1.76E-04	CDK1, MAD2L1, AURKA
	GO:0005654~nucleoplasm	6.99E-04	TOP2A, CCNA2, FANCI, CCNB1, RFC3, CDK1, AURKA
**CC**	GO:0031616~spindle pole centrosome	.004928796	DLGAP5, AURKA
	GO:0005813~centrosome	.017603051	CCNB1, CDK1, AURKA
	GO:0005876~spindle microtubule	.021525589	CDK1, AURKA
	GO:0005634~nucleus	.024083604	TOP2A, CCNA2, CCNB1, CDK1, DLGAP5, MAD2L1, AURKA

	**Term**	***P* value**	**Genes**
	hsa04914:Progesterone-mediated oocyte maturation	6.59E-05	CCNA2, CCNB1, CDK1, MAD2L1
**KEGG**	hsa04114:Oocyte meiosis	1.37E-04	CCNB1, CDK1, MAD2L1, AURKA
	hsa04110:Cell cycle	1.90E-04	CCNA2, CCNB1, CDK1, MAD2L1

### 3.4. Validation and survival analysis

**Figure 6.**
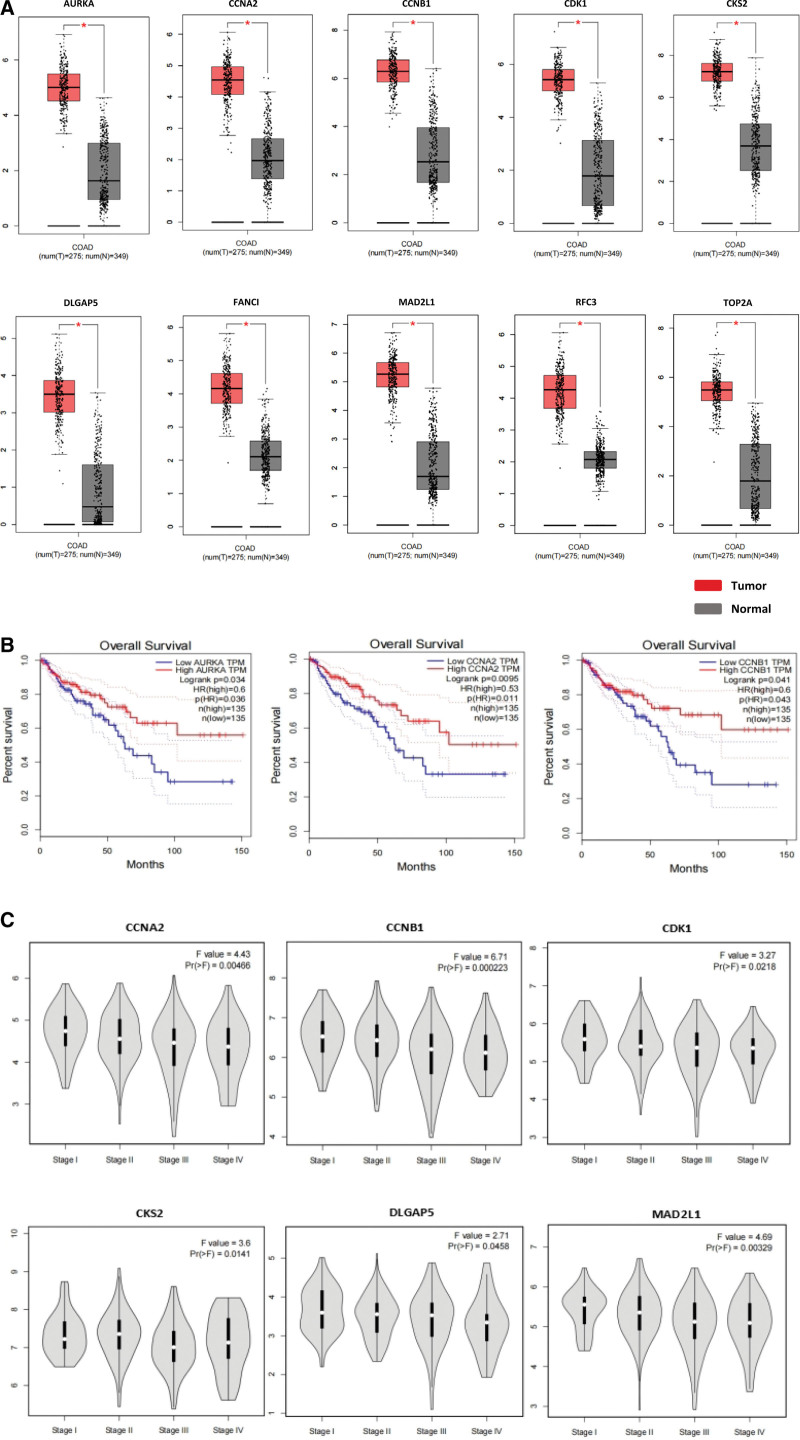
Gene expression, survival rate, and stage plot of key genes. (A) Gene expression of all screened key genes. T: tumor; N: normal. *P* < .05 was considered a significant difference (B) Correlation analysis of AURKA, CCNB1, and CCNA2 genes with regard to survival. (C) Correlation analysis of gene expression of CCNA2, CCNB1, CDK1, CKS2, DLGAP5, and MAD2L1 and their involvement in different CRC stages.

To further validate the plausibility of our screened key genes, we verified these 10 genes based on the TCGA database through GEPIA. We found that the mRNA expression levels of all the key genes in the tumor group were significantly higher than the normal tissue group (*P* < .05) (Fig. 6A). GEPIA was also used to calculate the survival rate which revealed that only AURKA, CCNB1, and CCNA2 were significantly associated with the survival of CRC patients (Fig. 6B). Moreover, we discovered that the CCNA2 (*P* = .00466), CCNB1 (*P* = .000223), CDK1 (*P* = .0218), CKS2 (*P* = .0141), DLGAP5 (*P* = .0458), and MAD2L1 (*P* = .00329) genes were differentially expressed in different stages of CRC (Fig. 6C).

## 4. Discussion

Early detection and the corresponding improvement in the prognosis for CRC patients is challenging although current clinical diagnosis and treatment methods show promising results. In the last few decades, many scientists have devoted their research careers to the revelation of the molecular mechanisms underlying CRC pathogenesis to be able to better identify CRC development and eventually provide more reliable methods for early clinical diagnosis and treatment.^[10]^

In the present study, we explored key genes and signaling pathways related to CRC that may increase our understanding of the potential molecular mechanisms and their benefits for the diagnosis, treatment, and prognosis of the disease. We searched for DEGs related to CRC occurrence and analyzed their gene expression profiles with GEO 2R. We then identified 10 key genes within the 120 DEGs through STRING and Cytoscape software that were tested with the help of the GEPIA and TCGA databases. In the end, we found that AURKA, CCNB1, and CCNA2 were significantly correlated with the overall survival of CRC patients whereas CCNA2, CCNB1, CDK1, CKS2, DLGAP5, and MAD2L1 could be correlated to the pathological stages of CRC.

Aurora Kinase A (AURKA) is a member of the serine/threonine protein kinase family which is involved in the assembly and repair of mitotic spindles. It can be autophosphorylated, can shuttle from the cytoplasm to the nucleus, and remains stable during the cell cycle.^[11,12]^ The activation of the serine/threonine kinase family is necessary to regulate mitotic cells. Its expression in different cancer tissues is significantly higher than that of normal tissues according to the TCGA database.^[13]^ It has been shown that increased AURKA expression in pancreatic cancer patients was negatively correlated with overall survival which may support the idea that AURKA constitutes a relevant therapeutic target for pancreatic ductal adenocarcinoma patients (PDAC).^[14]^ Moreover, Guo found that overexpression of AURKA promoted bladder cancer cell proliferation and predicted poor prognosis, implying that AURKA could be a valid biomarker for BC testing and prognosis, and possibly also as a target for treatment.^[15]^ Although several studies showed that AURKA is highly expressed in many types of tumor tissues and is directly correlated with the patient’s prognosis, no relevant research concerning whether AURKA plays a significant role in colorectal cancer or not has been conducted so far. Therefore, answering this question provides a new direction for future research.

It is well known that cancer cells can proliferate indefinitely and therefore, destroy the architecture and function of normal tissues. Many studies aiming at elucidating cell cycle regulation have been conducted, hoping that the generated knowledge might contribute to treating cancer more effectively. In particular, studies have shown that CCNB1 and CDK1 are important molecules involved in cell proliferation and cell cycle which were closely related to mitosis.^[16]^ Zou et al indicated that mRNA expression of CDK1 and CCNB1 was upregulated in hepatocellular carcinoma (HCC) and the higher the expression of these genes the poorer the prognosis of the HCC patients.^[17]^ High expression of CDK1 was also detected in lung cancer and pancreatic and colorectal cancer^[18–20]^ and was associated with the prognosis of the patients. Another gene regulating the cell cycle is CKS2 (cyclin-dependent kinase subunit 2). It plays an important role in the regulation of cell cycle progression since it is a regulatory subunit of CDK1.^[21]^ Numerous studies showed that dysregulation of CKS2 may contribute to the growth and metastasis of tumors in breast cancer, ovarian cancer, hepatocellular carcinoma, non-small-cell lung cancer, and colorectal cancer.^[22–26]^ CCNA2, CCNB1, CDK1, and CKS2 are key molecules involved in the cell cycle and play a crucial role in tumorigenesis and cancer progression. Their significance for the diagnosis and treatment of colorectal cancer is also well known.

DLGAP5, also known as discs large homolog 7 (DLG7) or hepatoma upregulated protein (HURP),^[27]^ is located in the spindle apparatus during mitosis. It is a mitotic phosphorylation protein regulated by the ubiquitin-proteasome pathway and a vital regulator of the integrity and differentiation of adhesion molecules.^[27]^ MAD2L1 is an important component of the spindle checkpoint and plays a role in overseeing chromosome segregation during mitosis.^[28]^ As key elements of the cell cycle, DLGAP5 and MAD2L1 are closely related to tumor development, especially in the spindle assembly which is one of the main development directions of chemotherapy.^[29]^

The main limitation of this research is that we harvested all results based on Gene Expression Omnibus (GEO) datasets, it would be better if we could further validation of the key genes in human colon cancer tissue. Nevertheless, we are working diligently to make an ethical application to obtain human colon cancer tissue samples to further demonstrate our point in our subsequent studies.

## 5. Conclusion

In summary, in this study, based on bioinformatics analysis, we have identified 7 key genes, namely AURKA, CCNA2, CCNB1, CDK1, CKS2, DLGAP5, and MAD2L1 from hundreds of genes and the momentous signaling pathways. AURKA, CCNB1, and CCNA2 are significantly related to the overall survival of CRC patients. CCNA2, CCNB1, CDK1, CKS2, DLGAP5, and MAD2L1 are correlated with the pathological CRC stage which means that they may be useful as new independent prognostic biomarkers in predicting the clinical treatment effect in CRC patients. These findings can provide us with a deeper understanding of the molecular mechanisms of CRC progression and may cater to potential biomarkers for the diagnosis, treatment, and prognosis of CRC patients in the future.

## Acknowledgment

The authors gratefully thank the support from China Scholarship Council.

## Author contributions

Conceptualization: Chaochao Wang.

Investigation: Chaochao Wang, Li Zhang.

Software: Chaochao Wang.

Supervision: Li Zhang.

Writing – original draft: Chaochao Wang.

Writing – review & editing: Li Zhang, Chaochao Wang.
